# Restraint Stress Alters Expression of Glucocorticoid Bioavailability Mediators, Suppresses Nrf2, and Promotes Oxidative Stress in Liver Tissue

**DOI:** 10.3390/antiox9090853

**Published:** 2020-09-11

**Authors:** Hsiao-Jou Cortina Chen, Tsz Yip, Johnny K. Lee, Juliani Juliani, Conrad Sernia, Andrew F. Hill, Nickolas A. Lavidis, Jereme G. Spiers

**Affiliations:** 1 School of Biomedical Sciences, The University of Queensland, St Lucia 4072, Australia; clintyip@gmail.com (T.Y.); johnny_k_lee@hotmail.com (J.K.L.); c.sernia@uq.edu.au (C.S.); lavidis@uq.edu.au (N.A.L.); 2 WT-MRC Institute of Metabolic Science, University of Cambridge, Cambridge CB2 0QQ, UK; 3 Department of Biochemistry and Genetics, La Trobe Institute for Molecular Science, La Trobe University, Bundoora 3083, Australia; 17833885@students.latrobe.edu.au (J.J.); andrew.hill@latrobe.edu.au (A.F.H.)

**Keywords:** antioxidant response, corticosteroids, liver, Nrf2, oxidative stress

## Abstract

Hepatic glutathione synthesis and antioxidant protection are critically important for efficient detoxification processes in response to metabolic challenges. However, this biosynthetic pathway, regulated by nuclear factor (erythroid-derived 2)-like 2 (Nrf2), previously demonstrated paradoxical repression following exposure to glucocorticoid stress hormones in cultured hepatic cells. Therefore, the present study used an in vivo model of sub-acute psychological stress to investigate the relationship between hepatic corticosteroid regulation and antioxidant systems. Male Wistar rats were kept under control conditions or subjected to six hours of restraint stress applied for 1 or 3 days (*n* = 8 per group) after which the liver was isolated for assays of oxidative/nitrosative status and expression of corticosteroid regulatory and Nrf2-antioxidant response element pathway members. A single stress exposure produced a significant increase in the expression of corticosterone reactivator, 11-beta-hydroxysteroid dehydrogenase 1 (11β-Hsd1), while the 11β-Hsd2 isozyme and corticosteroid-binding globulin were down-regulated following stress, indicative of an elevated availability of active corticosterone. Exposure to restraint significantly decreased hepatic concentrations of total cysteine thiols and the antioxidant reduced glutathione on Day 1 and increased 3-nitrotyrosinated and carbonylated proteins on Day 3, suggestive of oxidative/nitrosative stress in the liver following stress exposure. Conversely, there was a sustained down-regulation of Nrf2 mRNA and protein in addition to significant reductions in downstream glutamate-cysteine ligase catalytic subunit (Gclc), the rate-limiting enzyme in glutathione synthesis, on Day 1 and 3 of stress treatment. Interestingly, other antioxidant genes including superoxide dismutase 1 and 2, and glutathione peroxidase 4 were significantly up-regulated following an episode of restraint stress. In conclusion, the results of the present study indicate that increased expression of 11β-Hsd1, indicative of elevated tissue glucocorticoid concentrations, may impair the Nrf2-dependent antioxidant response.

## 1. Introduction

Glucocorticoids (cortisol and corticosterone) are the main effector hormones of the hypothalamic-pituitary-adrenal (HPA) axis and their levels dynamically increase with exposure to physiological and psychological stressors. Termination of the stress response is mediated by glucocorticoids via negative feedback to the anterior pituitary, hypothalamus, and extra-hypothalamic centers which reduces HPA axis flux to restore homeostasis [1,2]. According to the free hormone hypothesis, this feedback is mediated by the unbound, physiologically active glucocorticoids that are able to cross the blood-brain barrier and bind glucocorticoid receptors [3]. It was estimated that about 5% of glucocorticoids circulate freely, while 80–90% bind to corticosteroid-binding globulin (CBG; formally transcortin), and 5–10% bind non-specifically to albumin in the bloodstream [4,5]. Previous reports demonstrated that CBG, produced primarily in the liver, plays an important role in regulation of the stress response, with reductions in CBG expression transiently increasing free glucocorticoid bioavailability [6,7,8,9]. The action of glucocorticoids on target tissues is mediated by glucocorticoid (GR) and mineralocorticoid (MR) receptors which exert canonical nuclear hormone receptor effects and rapid non-genomic membrane-associated activity [10]. In addition to circulating concentrations, local tissue levels of glucocorticoids can be modulated by two isozymes of 11β-hydroxysteroid dehydrogenase (11β-HSD) [11]. The type one 11β-HSD (11β-Hsd1) isozyme predominantly possesses reductase activity and catalyzes the NADPH-dependent conversion of inert cortisone and 11-dehydrocorticosterone to active cortisol and corticosterone, respectively [12]. We previously showed that an up-regulation of 11β-Hsd1 expression in extra-hypothalamic regions may facilitate negative feedback suppression of the HPA axis following acute stress [13]. The second isozyme (11β-Hsd2) requires NAD^+^ as co-factor and exclusively possesses dehydrogenase activity which acts to rapidly inactivate glucocorticoids by converting cortisol/corticosterone back to their respective keto-forms [14]. A critical role of 11β-Hsd2 is to protect the MR from promiscuous activation by promoting glucocorticoid metabolism, thereby safeguarding the activity of aldosterone as the principal physiological agonist of MR in aldosterone-sensitive regions [15,16].

Several animal studies reported substantial hepatocellular damage in the form of elevated liver enzymes following acute and chronic stress [17,18,19,20]. This is often accompanied by increased markers of oxidative stress, including increased oxidized glutathione (GSSG), lipid peroxidation, nitrite and nitrate (NO_x_), and myeloperoxidase activity in the liver following stress [17,19,21]. This stress-induced imbalance in reduction/oxidation (redox) state is simultaneously observed with decreased antioxidant components including reduced glutathione (GSH), superoxide dismutase (SOD), catalase, and ascorbic acid [21,22]. A major mechanism in the cellular defense against oxidative stress is activation of nuclear factor erythroid 2 (NFE2)-related factor 2 (Nrf2). Following nuclear translocation, activated Nrf2 binds to antioxidant response elements (ARE) to regulate phase II detoxifying enzymes such as glutamate-cysteine ligase, glutathione peroxidase (GPx), heme oxygenase 1 (Hmox1), and quinone oxidoreductase 1 (Nqo1) [23]. Studies using Nrf2 knockout mice demonstrated substantial susceptibility to a broad range of chemical toxicities and disease conditions associated with oxidative pathology [24,25,26]. Interestingly, work by Kratschmar and colleagues [27] showed that hepatic Nrf2 is suppressed in response to increased activity of 11β-Hsd1 in vitro. However, the relationship between hepatic corticosteroid regulation and antioxidant systems following psychological stress in vivo has not been well characterized. The present study used an in vivo model of sub-acute psychological stress to investigate the relationship between hepatic corticosteroid regulation and antioxidant systems following stress exposure.

## 2. Materials and Methods

### 2.1. Animals

Ethics approval was obtained from the University of Queensland Animal Ethics Committee under approval number SBS/456/14/URG. Male Wistar rats (298.3 ± 4.1 g) aged 7–8 weeks were bred and sourced from The University of Queensland Biological Resources and housed in groups of four within the Australian Institute of Biotechnology and Nanotechnology animal facility. Rats were housed in a colony room with controlled temperature (22 ± 2 °C) and humidity; (55 ± 5%) on a 12-h light-dark cycle (lights on at 07.00 h) with food and water provided *ad libitum*. Rats were habituated to handling for 10 min per day over six consecutive days prior to experimentation.

### 2.2. Experimental Protocol

Rats were randomly allocated into treatment groups of no stress (unstressed; US), acute (1 Day, single episode of 6 h), or repeated restraint stress (3 Days, 6 h per day) from 9.00 h to 15.00 h using adjustable wire mesh restrainers (*n* = 8 per group). Rats were transported on each treatment day to an experimental room within the same animal facility and were acclimated for 1 h prior to any experimental procedures. At the end of the treatment, rats were immediately anesthetized with Pentobarbital Sodium (100 mg/kg, intraperitoneal injection; Lethabarb, Virbac) and whole blood samples were collected via cardiac puncture into ice-chilled tube containing heparin (20 IU/mL blood). Blood samples were centrifuged at 2000× *g* and the resultant plasma was collected and stored at −80 °C for measurement of total corticosterone levels. Liver tissues were quickly snap-frozen for later relative mRNA expression, biochemical, Western blotting, and immunofluorescence analysis.

### 2.3. Plasma Corticosterone Measurements

Total plasma corticosterone concentrations were determined using DetectX^®^ Corticosterone Enzyme Immunoassay Kit (Cat. No. K014-H1; Arbor Assays^TM^, Ann Arbor, MI, USA) according to the manufacturers’ instructions.

### 2.4. mRNA Expression

For the analysis of gene expression, total RNA was isolated from liver tissues using the RNeasy mini kit (QIAGEN, Doncaster, Australia) and further purified with deoxyribonuclease 1 treatment (QIAGEN) according to the supplier’s protocol. Extracted RNA concentrations and purity were measured using the Nanodrop 2000c spectrophotometer and Agilent 2100 Bioanalyzer respectively. Agilent RNA 6000 Nano Kits were used to determine the RNA integrity values, with an average RIN = 9.23 for all samples analyzed. Subsequently, 2 µg of RNA was reverse transcribed into cDNA using the iScript^TM^ reverse transcription supermix, including controls omitting the reverse transcriptase (Bio-Rad Laboratories, Gladesville, Australia). The cDNA was amplified using gene specific TaqMan^TM^ Gene Expression Assays (Applied Biosystems, Foster City, CA, USA) for the genes listed in Table 1. No amplification was observed in the template omission or reverse transcriptase omission controls. Target gene expression was determined relative to a primer-limited and well-characterized VIC-labeled β-actin (*Actb*; Applied Biosystems) using the formula 2-ΔCt where ΔCt = (Ct target gene − Ct ACTB) and expressed relative to unstressed controls. No changes were observed in target gene amplification efficiency using this multiplex reaction system.

### 2.5. Preparation of Liver Tissue for Biochemical Assays

Biochemical assays of oxidative and nitrosative status were performed in isolated liver tissues homogenized (IKA^®^ Ultra-Turrax^®^ T10) in 7.5 volumes (*w*/*v*) of Tris-HCl (0.05 M, pH = 7.4) containing a cocktail of protease inhibitors (Cat no S8830, Sigma-Aldrich, Castle Hill, Australia). An aliquot of liver crude homogenate was removed for GSH and GSSG determination. This aliquot was loaded with an acidic solution containing 10% trichloroacetic acid (*w*/*v*) in 0.1 M potassium phosphate buffer containing 5 mM EDTA (KPE buffer; pH = 7.5) and left on ice for 5 min prior to centrifugation at 10,000× *g* for 5 min to prevent the oxidation of reduced GSH. The remaining crude homogenate was centrifuged at 10,000× *g* for 10 min at 4 °C and the supernatant was used for the determination of free cysteine thiols, general oxidative status, protein carbonylation, lipid peroxidation, and 3-nitrotyrosine with corresponding protein concentrations.

### 2.6. Reduced and Oxidized Glutathione

Reduced GSH and GSSG concentrations were determined as described previously by our group [13] with slight modifications. Liver homogenates deproteinated with trichloroacetic acid were further treated with or without N-ethylmaleimide and redox quenched samples were loaded with the fluorophore O-phthaldialdehyde (OPA). Samples were incubated for 30 min in the dark and fluorescence was determined using a POLARstar OPTIMA (BMG Labtechnologies, Offenburg, Germany) with excitation and emission filters set at 365 nm and 430 nm, respectively. GSH and GSSG levels were subsequently adjusted to corresponding total protein concentrations, and expressed as percentage change relative to unstressed values.

### 2.7. Free Thiol Detection

The Thiol Detection Assay Kit (Cat no: 700340, Cayman Chemical, Ann Arbor, MI, USA) was used to determine liver free thiol content according to the manufacturer’s instructions and presented as nM per mg protein.

### 2.8. General Oxidative Status

General oxidative status was determined in liver samples as described in our previous work [28] with slight modifications. A working solution of 100 μM 2′,7′-dichlorofluorescein diacetate (DCFH-DA) was prepared in 0.05 M of Tris-HCl. Diluted supernatant of liver homogenate was loaded with 50 μL of 100 μM DCFH-DA and incubated for 30 min at 37 °C. Sample fluorescence was measured by POLARstar^®^ OPTIMA microplate reader (BMG Labtechnologies) with excitation and emission filters set at 485 nm and 520 nm respectively. Data was normalized with corresponding protein concentrations and expressed as arbitrary fluorescence units (AU) per mg protein.

### 2.9. Lipid Peroxidation

The peroxidation of lipid in hepatic tissue was determined using a thiobarbituric acid-reactive-substances (TBARS) assay as described in our previous work [29]. The reacted malondialdehyde (MDA)-thiobarbituric (TBA) adduct in the supernatant of liver homogenate was extracted with n-butanol and measured fluorometrically at 544 nm (excitation) and 590 nm (emission) using the POLARstar OPTIMA microplate reader. Sample values were quantified from a standard curve using 1,1,3,3-tetraethoxypropane as an external standard, corrected for corresponding protein concentrations, and expressed as nM per mg of protein.

### 2.10. Protein Carbonyl

Protein-bound carbonyls were determined via a protein carbonyl assay kit (Cat no: 10005020, Cayman Chemical). The assay uses the covalent reaction of 2,4-dinitrophenylhydrazine with the carbonylated protein side chain and the amount of protein–hydrazone produced is quantified spectrophotometrically at 370 nm. The results were calculated using the extinction coefficient of 22 mM^−1^cm^−1^ for aliphatic hydrazones, corrected for corresponding protein concentrations, and expressed as nmol per mg protein.

### 2.11. 3-Nitrotyrosine

Quantification of 3-nitrotyrosine modified proteins in liver supernatant was performed using a commercially available ELISA kit (Cat no: ab116691, Abcam, Cambridge, MA, USA). Unknown concentrations were determined from 3-NT bovine serum albumin standard equivalents, corrected for corresponding protein concentrations, and expressed as ng per mg protein.

### 2.12. Immunofluorescence

Snap-frozen liver tissues were cut into 12 μm sections, mounted, washed three times in phosphate-buffered saline, and subsequently fixed for 15 min with phosphate-buffered paraformaldehyde (4%, *w*/*v*). Citrate antigen retrieval was performed for 20 min at 95 °C prior to blocking in normal goat serum and overnight incubation with primary antibodies against Sod1 (1:200 dilution; Cat no: ADI-SOD-100-D, Enzo Life Sciences, Plymouth Meeting, PA), 4-hydroxy-2-nonenal (4-HNE; 1:200 dilution; Cat no: ab46545, Abcam), and Nrf2 (1:50 dilution; Cat no: NBP1-32822, Novus Biologicals, Littleton, CO, USA) at 4 °C. Secondary antibodies (1:500 dilution; Alexa Fluor^TM^ 488 goat anti-rabbit, Invitrogen, Mt Waverley, VIC, Australia) were subsequently applied and sections were counterstained with Hoechst 33342 (1:2000 dilution; ThermoFisher Scientific, San Diego, CA, USA). Sections were imaged at 20× magnification and at 63× for NRF2 nuclear localization under confocal microscopy (Zeiss LSM 780) and ImageJ was used for further analysis of Sod1, 4-HNE, and Nrf2 immunofluorescence.

### 2.13. Western Blotting

Liver tissues were prepared and lyzed using a buffer containing 20 mM Tris (pH 7.5), 1% Triton X-100 (*v*/*v*), and protease inhibitors. The protein concentration of each sample was determined by the bicinchoninic acid method and equal amounts of protein (20 μg) were separated by electrophoresis on Bolt™ 4 to 12% Mini Protein Gels (ThermoFisher). After electrophoresis, the proteins were transferred to polyvinylidene difluoride membranes, followed by nonspecific blocking (5% *w*/*v* skimmed milk in phosphate-buffered saline with 0.05% *v*/*v* Tween^®^ 20) for 1 h at room temperature. The membranes were then incubated with primary antibodies against Sod1 (1:1000 dilution; Cat no: ADI-SOD-100-D, Enzo Life Sciences) and Nrf2 (1:500 dilution; Cat no: NBP1-32822, Novus Biologicals) overnight at 4 °C. After 4–5 washes, the membranes were treated with the appropriate secondary antibodies (1:15,000 dilution; IRDye^®^ 680RD and IRDye^®^ 800CW Infrared Dyes, Li-Cor, Lincoln, NE) for 1 h. β-actin (1:3000 dilution; Cat no: ab6276, Abcam) was used as the internal loading control for all of the Western blotting experiments. The blots were visualized using the Odyssey^®^ CLx Infrared Imaging System (Li-Cor).

### 2.14. Protein Determination

Protein was determined using commercially available Red660^TM^ protein kit (G-Biosciences, St. Louis, MO, USA) using bovine serum albumin as a standard.

### 2.15. Statistical Analyses

All data were expressed as mean ± standard error of the mean (±SEM). Statistical comparisons were performed using GraphPad Prism (Version 8.0.1, GraphPad Software Inc., San Diego, CA, USA) with *p* ≤ 0.05 considered to be statistically significant. Data were first analyzed for normality using the Brown-Forsythe test. One-way ANOVA with Fisher’s least significant difference (LSD) test was used to compare normally distributed data. Non-parametric Kruskal-Wallis ANOVA with Dunn’s multiple comparisons test was used for data sets with significantly different standard deviations.

## 3. Results

### 3.1. Restraint Stress-Induced Corticosterone Was Accompanied by Altered Hepatic Expression of Glucocorticoid Bioavailability Mediators

**Figure 1:**
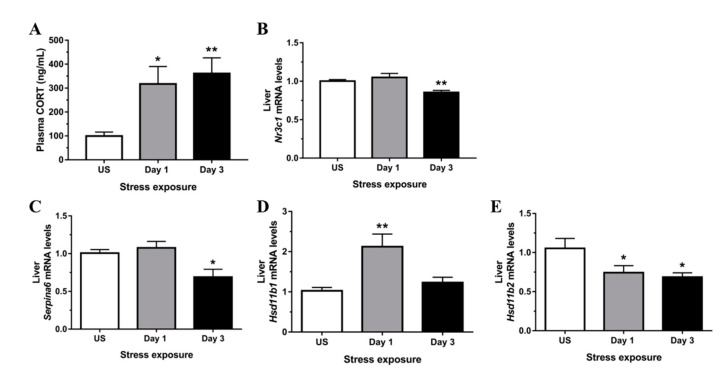
Restraint stress (**A**) increases circulating corticosterone (CORT), (**B**) reduces expression of glucocorticoid receptor (*Nr3c1*) and modulates mRNA expression of hepatic mediators of glucocorticoid bioavailability including (**C**) corticosteroid-binding globulin (CBG; *Serpina6*), (**D**) hydroxysteroid 11-beta dehydrogenase 1 (*Hsd11b1*), and (**E**) hydroxysteroid 11-beta dehydrogenase 2 (*Hsd11b2*) compared to unstressed (US) rats (*n* = 6–8/group). Results in (**C**,**E**) were analyzed using one-way ANOVA with Fisher’s LSD test; (**A**,**B**,**D**) were analyzed using non-parametric Kruskal-Wallis test with Dunn’s post-test. Data are expressed as mean ± SEM, * *p* < 0.05 and ** *p* < 0.01.

Restraint stress significantly increased plasma corticosterone above values of unstressed controls following a single episode (*p* < 0.05) and repeated restraint stress (*p* < 0.01) exposure (Figure 1A). The availability of GR governs subsequent corticosteroid responsiveness, and in the present study, mRNA expression of GR (*Nr3c1*) in the liver was significantly down-regulated following 3 days (*p* < 0.01) of repeated stress (Figure 1B). The negative acute phase protein, CBG, is synthesized and secreted by the liver to modulate tissue availability of glucocorticoids. In the present study, stress treatment significantly down-regulated hepatic expression of CBG (*Serpina6*) following 3 days (*p* < 0.05) of repeated restraint (Figure 1C). The 11β-Hsd1 enzyme converts inactive glucocorticoids into active corticosterone, and stress significantly increased *Hsd11b1* mRNA levels following 1 day (*p* < 0.01) of stress relative to unstressed controls (Figure 1D). Concurrently, *Hsd11b2* mRNA expression was significantly down-regulated following both day 1 and 3 (*p* < 0.05) of stress treatment (Figure 1E). Together, increased 11β-Hsd1 and decreased 11β-Hsd2 expression indicates an increased capacity for regeneration of active glucocorticoids locally in hepatic tissue.

### 3.2. Restraint Stress-Induced Hepatic Oxidative and Nitrosative Imbalance and Oxidative Damage

**Figure 2:**
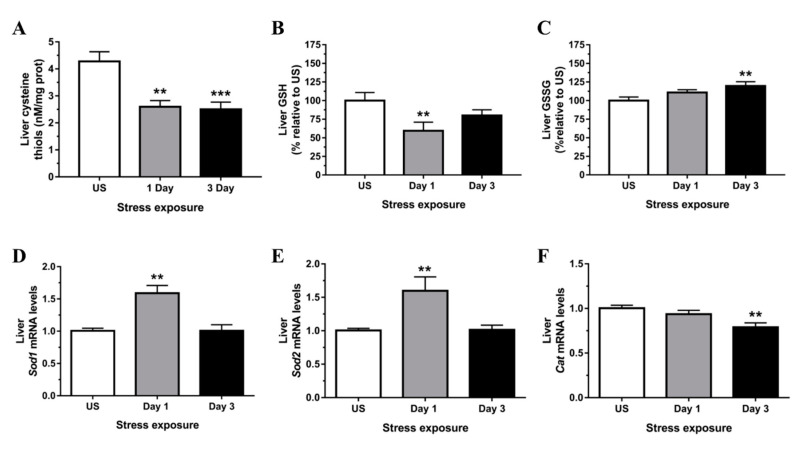
Restraint stress induces hepatic redox imbalance as shown by (**A**) decreased free cysteine thiol content and (**B**) decreased reduced glutathione (GSH) with increased (**C**) glutathione disulfide (GSSG). This occurs alongside increased mRNA expression of (**D**) superoxide dismutase 1 (*Sod1*) and (**E**) superoxide dismutase 2 (*Sod2*) but decreased (**F**) catalase (*Cat*) compared to unstressed (US) rats (*n* = 7–8/group). Results in (**A**–**C**,**F**) were analyzed using one-way ANOVA with Fisher’s LSD test; (**D**,**E**) were analyzed using non-parametric Kruskal-Wallis test with Dunn’s post-test. Data are expressed as mean ± SEM, ** *p* < 0.01, *** *p* < 0.001.

In the present study, we used established biochemical and molecular indicators to demonstrate the profile of redox changes within the liver following acute and repeated restraint stress. Free thiols are essential antioxidant mediators with the capacity to maintain redox homeostasis and include thiol/disulfide redox couples such as 2GSH/GSSG, thioredoxins, and other cysteine-containing proteins. As shown in Figure 2A, free cysteine thiol content in the liver was significantly reduced following 1 (*p* < 0.01) and 3 (*p* < 0.001) days of restraint stress. Moreover, stress exposure significantly reduced levels of hepatic GSH (Figure 2B) and increased levels of hepatic GSSG (Figure 2C). Together, these results suggest that acute and repeated stress induces a pro-oxidative state through a loss in GSH/GSSG redox balance in the liver.

**Figure 3:**
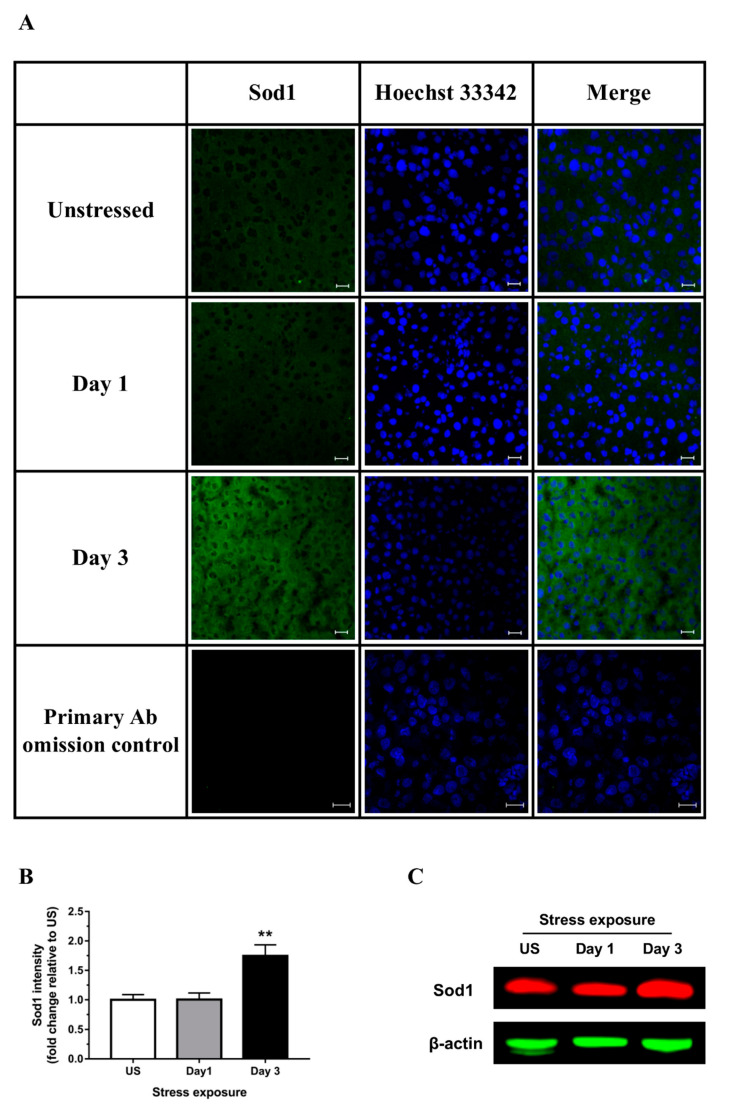
Representative (**A**) immunofluorescence images (scale bar = 20 μm), (**B**) corresponding image quantification (*n* = 3), and (**C**) Western blot of hepatic superoxide dismutase 1 (Sod1). Results in (**B**) were analyzed using one-way ANOVA with Fisher’s LSD test. Data are expressed as mean ± SEM, ** *p* < 0.01.

Superoxide dismutase, a key cellular antioxidant, acts to enzymatically eliminate superoxide radicals (O_2_^−^). The mRNA expression of copper-zinc superoxide dismutase (*Sod1*; Cu, Zn-SOD) and manganese-dependent superoxide dismutase (*Sod2*; Mn-SOD) were both up-regulated in the liver following 1 day (*p* < 0.01) of stress (Figure 2D,E). As shown in Figure 3A,B there was also a significant increase in Sod1 signal within the liver following 3 days (*p* < 0.01) of restraint stress. A similar protein expression pattern of Sod1 was demonstrated by Western blot (a representative blot is shown in Figure 3C). Even though the antioxidant activity of catalase was suggested to be highest in the liver to eliminate H_2_O_2_, we observed a significant down-regulation of liver catalase (*Cat*) mRNA expression following 3 days (*p* < 0.01) of repeated restraint stress when compared to unstressed controls (Figure 2F).

**Figure 4:**
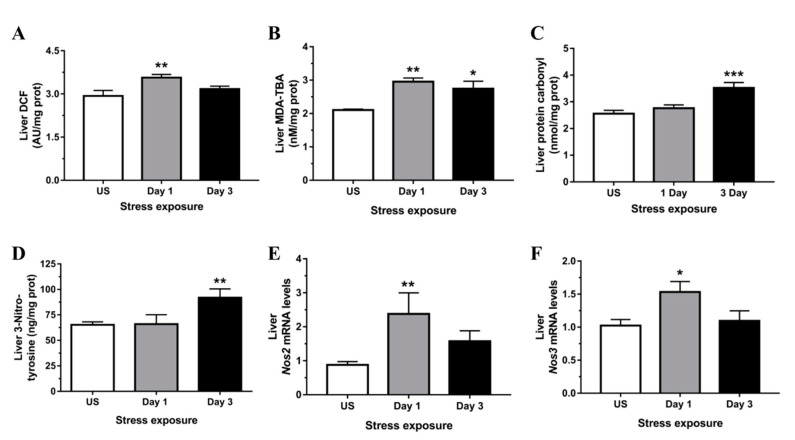
Restraint stress increases hepatic oxidative/nitrosative stress indicated by (**A**) dichlorofluorescein (DCF) formation, (**B**) malondialdehyde-thiobarbituric acid (MDA-TBA) adduct, (**C**) protein carbonyl, (**D**) 3-nitrotyrosine, and both (**E**) nitric oxide synthase 2, inducible (*Nos2*), and (**F**) nitric oxide synthase 3, endothelial cell (*Nos3*) expression compared to unstressed (US) rats (*n* = 7–8/group). Results in (**A**,**C**,**F**) were analyzed using one-way ANOVA with Fisher’s LSD test; (**B**,**D**,**E**) were analyzed using non-parametric Kruskal-Wallis test with Dunn’s post-test. Data are expressed as mean ± SEM, * *p* < 0.05, ** *p* < 0.01, *** *p* < 0.001.

**Figure 5:**
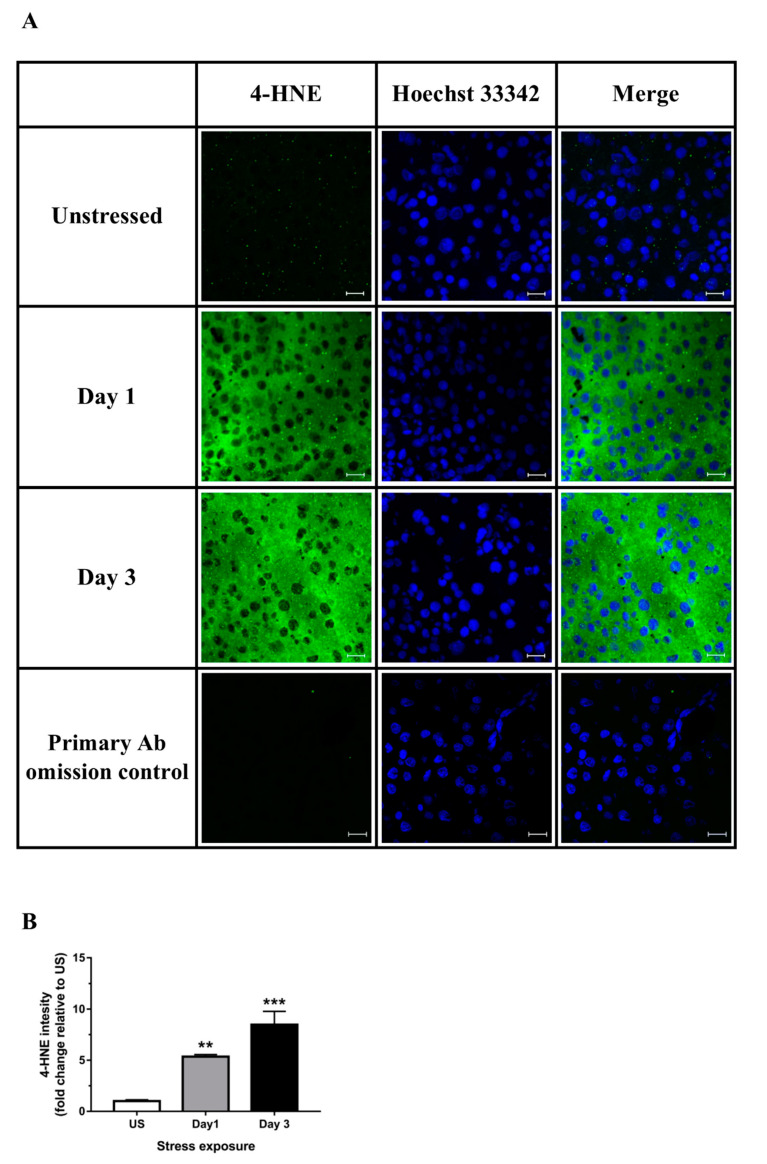
Representative images of (**A**) hepatic 4-hydroxynonenal (4-HNE; scale bar = 20 μm) and (**B**) corresponding image quantification (*n* = 3). Results in (**B**) were analyzed using one-way ANOVA with Fisher’s LSD test. Data are expressed as mean ± SEM, ** *p* < 0.01, *** *p* < 0.001.

The effects of stress on the general oxidative status in the liver was determined using DCFH-DA. The oxidation of H_2_DCF to DCF was highest following 1 day (*p* < 0.01) of acute stress (Figure 4A). Stress was also highly effective at increasing hepatic peroxidation of lipids following both acute (*p* < 0.01) and repeated (*p* < 0.05) stress (Figure 4B). Furthermore, we found a significant increase in immunoreactivity for 4-HNE, a major cytotoxic end product of lipid peroxidation, following 1 (*p* < 0.01) and 3 (*p* < 0.001) days of restraint stress in the liver (Figure 5A,B). Protein carbonylation represents an irreversible post-translational modification formed during oxidative stress conditions. We found that stress significantly increased hepatic carbonylated proteins following 3 days (*p* < 0.001) of repeated stress (Figure 4C).

We further found that the level of 3-nitrotyrosine was significantly increased in the liver following 3 days (*p* < 0.01) of repeated restraint stress (Figure 4D). Peroxynitrite forms through the reaction of nitric oxide (NO) with O_2_^−^; and expression of both NO-catalyzing enzymes abundantly present in the liver, inducible (*Nos2*; *p* < 0.01) and endothelial (*Nos3*; *p* < 0.05) nitric oxide synthase, was up-regulated following 1 day of stress exposure (Figure 4E,F).

### 3.3. Nrf2 Was Robustly Down-regulated Following Short-Term Stress in the Liver

**Figure 6:**
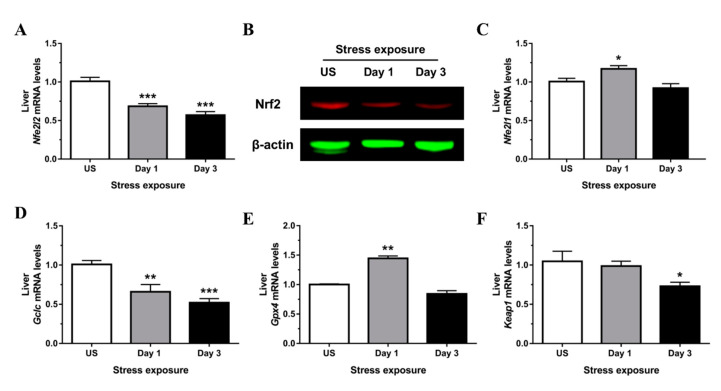
Restraint stress modulates hepatic (**A**) mRNA and (**B**) protein expression of nuclear factor, erythroid 2-like 2 (Nrf2) and other key antioxidant regulators including (**C**) nuclear factor, erythroid 2-like 1 (*Nrf1*), (**D**) glutamate-cysteine ligase, catalytic subunit (*Gclc*), (**E**) glutathione peroxidase 4 (*Gpx4*), and (**F**) Kelch-like ECH-associated protein 1 (*Keap1*) mRNA expression compared to unstressed (US) rats (*n* = 7–8/group). Results in (**A**–**D**) were analyzed using one-way ANOVA with Fisher’s LSD test; (**E**,**F**) were analyzed using non-parametric Kruskal-Wallis test with Dunn’s post-test. Data are expressed as mean ± SEM, * *p* < 0.05, ** *p* < 0.01, *** *p* < 0.001.

**Figure 7:**
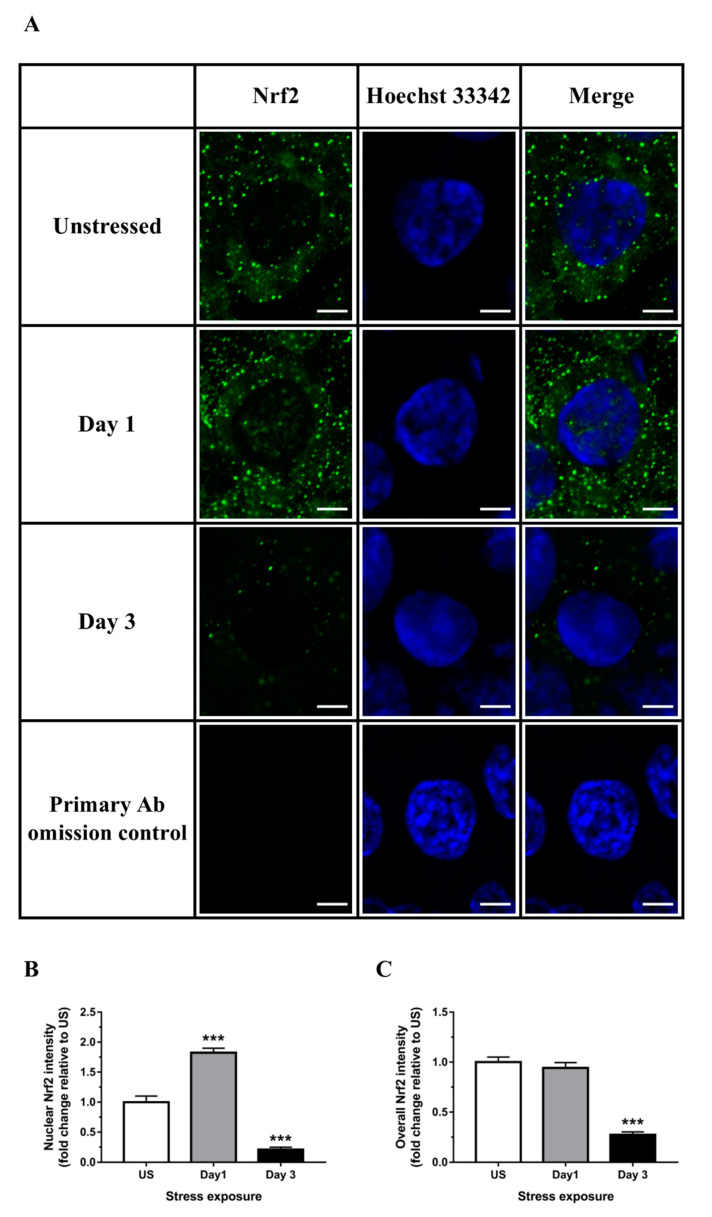
Representative (**A**) immunofluorescence images (scale bar = 5 μm) and image quantification of (**B**) corresponding nuclear Nrf2, and (**C**) overall Nrf2 from tissue sections in the liver (*n* = 3). Results in (**B**) were analyzed using one-way ANOVA with Fisher’s LSD test. Data are expressed as mean ± SEM, *** *p* < 0.001.

Despite a pro-oxidative state following stress, expression of Nrf2 (*Nfe2l2*), the master regulator of an array of detoxifying and antioxidant defense genes, was strongly down-regulated following both stress time-points (*p* < 0.001) in the liver (Figure 6A). Down-regulation of Nrf2 protein expression following 3 days of stress was further demonstrated using immunoblotting (Figure 6B) and confirmed by immunofluorescence analysis of nuclear localization and whole tissue sections (Figure 7A–C). Analogous to Nrf2, nuclear factor erythroid-2–related factor 1 (Nrf1; *Nfe2l1*) is also known to be essential for maintaining redox homeostasis and coordinating cellular stress responses. The expression of *Nfe2l1* in the liver was transiently up-regulated following a single episode of stress (Figure 6C). Some of the classical Nrf2 target genes involved in antioxidant defense include enzymes responsible for GSH synthesis (*Gclc*, glutamate-cysteine ligase catalytic subunit, and glutathione synthetase) and reduction of hydrogen peroxide (GPx family of enzymes). We found that expression of *Gclc*, the rate-limiting enzyme responsible for GSH production, was significantly reduced in the liver following 1 (*p* < 0.01) and 3 (*p* < 0.001) days of restraint stress when compared to unstressed controls (Figure 6D). Furthermore, glutathione peroxide 4 (*Gpx4*) was significantly up-regulated following one (*p* < 0.01) episode of stress exposure (Figure 6E). Interestingly, hepatic expression of Kelch-like ECH-associated protein 1 (*Keap1*), the specific repressor responsible for Nrf2 sequestration in the cytoplasm, was down-regulated following 3 days (*p* < 0.05) of stress (Figure 6F).

### 3.4. Phase II Detoxifying Enzymes Including Hmox1 and Nqo1 Were Up-regulated Following Restraint Stress

**Figure 8:**
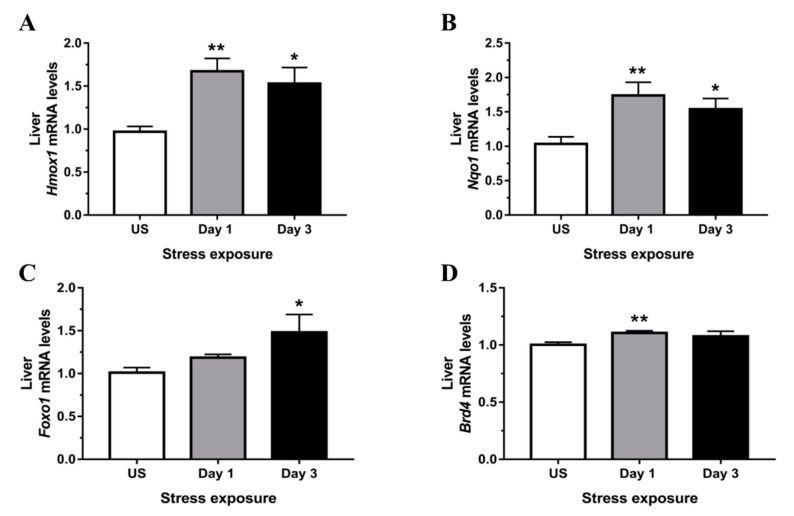
Restraint stress increases expression of Phase II detoxifying enzymes and alternative redox modulators including (**A**) heme oxygenase 1 (*Hmox1*), (**B**) NAD(P)H dehydrogenase, quinone 1 (*Nqo1*), (**C**) forkhead box O1 (*Foxo1*), and (**D**) bromodomain-containing protein 4 (*Brd4*) mRNA expression compared to unstressed (US) rats (*n* = 7–8/group). Results in (**A**,**B**) were analyzed using one-way ANOVA with Fisher’s LSD test; (**C**,**D**) were analyzed using non-parametric Kruskal-Wallis test with Dunn’s post-test. Data are expressed as mean ± SEM, * *p* < 0.05 and ** *p* < 0.01.

One of the most extensively studied phase II detoxifying/antioxidant enzymes, Hmox1, is an essential enzyme in heme catabolism, cleaving heme to release carbon monoxide, ferrous iron (Fe^2+^), and biliverdin. Following both 1 (*p* < 0.01) and 3 (*p* < 0.05) days of stress treatment, the mRNA expression of *Hmox1* was up-regulated in the liver (Figure 8A). Similarly, another Nrf2 target gene, *Nqo1*, was up-regulated following both 1 day (*p* < 0.01) and 3 days (*p* < 0.05) of stress relative to unstressed controls (Figure 8B). Figure 8C shows a significant up-regulation in Forkhead box protein O1 (*Foxo1*) mRNA expression, an alternative mediator of the Keap1/Nrf2/ARE pathway, following 3 days (*p* < 0.05) of repeated stress exposure. Finally, the mRNA expression of the epigenetic sensor, bromodomain-containing protein 4 (*Brd4*), known to regulate the Nrf2/Keap1 axis, showed a minor, albeit significant, up-regulation following 1 day (*p* < 0.01) of restraint stress (Figure 8D).

## 4. Discussion

The present study used a model of acute repeated restraint stress to investigate the relationship between high levels of glucocorticoids, redox balance, and the Nrf2-dependent and independent antioxidant responses in the liver. We showed that stress modulates local mediators of glucocorticoid bioavailability, including CBG and 11β-HSDs, reduces cellular antioxidant defense capacity likely via suppression of Nrf2-mediated GSH production, and induces an overall hepatic oxidative and nitrosative stress. This is in agreement with previous study by Kratschmar and colleagues [27], using cultured hepatic cells, which demonstrated that Nrf2 expression shared an inverse relationship with 11β-Hsd1, with an increase in the latter resulting in an impaired cellular oxidant neutralization response.

The observed elevation in circulating corticosterone at both stress time-points was accompanied by a down-regulation of GR (*Nr3c1*) and CBG (*Serpina6*) following 3 days of repeated acute stress. Fluctuations in CBG levels can directly affect glucocorticoid bioavailability through its property as a high-affinity corticosteroid-binding protein. Previous studies demonstrated down-regulation in CBG expression increases the free (unbound) fraction of glucocorticoids following both acute and chronic stressors [7,8]. This down-regulation is regulated by glucocorticoids at a transcriptional level via the GR which leads to C/EBPβ recruitment to the *Cbg* promoter [30]. The availability of tissue glucocorticoids is also governed via intracellular metabolism by 11β-HSDs. Although it was reported that 11β-Hsd1 can possess bidirectional enzymatic activity, the increase in its expression in vivo is believed to function as a reductase to generate active glucocorticoid, thus enhancing GR activation [31,32]. While 11β-Hsd2 acts exclusively as a dehydrogenase, the observed reduction in the present study following both stress time-points suggests there is reduced capacity to deactivate corticosterone [33]. Taken together, increased total circulating corticosterone and increased expression of 11β-Hsd1 with reduced expression of 11β-Hsd2 and CBG suggest that local hepatic tissues experience high levels of bioactive glucocorticoid resulting in GR down-regulation following stress.

As the end product of the HPA axis, glucocorticoids regulate multiple aspects of energy metabolism in the adaptive response to stress, including hepatic gluconeogenesis [34]. However, during the course of energy production, stress-induced glucocorticoids have been shown to be a causative factor of oxidative stress, inflammation, and damage within the liver [35]. Consistent with this observation, we showed a decrease in GSH and an increase in the product of GSH oxidation (GSSG), following 1 and 3 days of restraint stress, indicative of hepatic oxidative stress. There was also a sustained stress-induced decrease in free cysteine thiols, the immediate precursor of GSH, which further suggests an altered glutathione redox balance. Interestingly, the delayed increase in oxidized GSSG despite significant reductions in GSH and cysteine thiols suggests that GSSG formation may be secondary to other reactions that remove GSH from the measurable thiol pool. A likely candidate would be S-glutathionylation, a post-translational protein modification that was shown to increase in response to oxidative stress in primary rat hepatocytes and is involved in approximately 10% of 4-HNE disposition in the rat liver [36,37]. Additionally, Yu and Long [38] demonstrated that cysteine is the primary amino acid regulating cellular GSH homeostasis using hepatocellular carcinoma cells. Prior studies also demonstrated that a deficiency in intracellular cysteine can trigger a unique iron-dependent form of nonapoptotic cell death, termed ferroptosis, primarily due to failure in synthesizing GSH [39,40]. The expression profile of other antioxidant enzymes including Cu, Zn-SOD and mitochondrial Mn-SOD were also examined, with both becoming up-regulated following 6 hours of restraint, while hepatic catalase was down-regulated following 3 days of acute repeated stress. This is in agreement with a previous study employing longer stress durations where both immobilization and cold stress led to increased Cu, Zn-SOD and decreased catalase activities in the rat liver [41].

Further evidence that stress treatment induced liver redox imbalance were the increases in the general oxidative status, markers of lipid peroxidation, and protein oxidation in the liver of rats subjected to restraint stress. In agreement with the present study, Rajaraman and colleagues [42] showed that treatment with the synthetic glucocorticoid, dexamethasone, significantly increased DCF fluorescence together with higher lactate dehydrogenase release, indicating a general increase in reactive oxygen species and hepatocellular damage. As one of the mechanisms of stress-induced liver injury, lipid peroxidation was examined in the present study by both MDA formation and immunohistochemical staining for 4-HNE. Studies demonstrated that accumulation of lipid hydroperoxides induces ferroptosis driven by GSH depletion and/or loss of lipid-protective GPx4 enzyme activity [40,43]. Dodson and colleagues [44] provided further evidence that Nrf2 plays a pivotal role in preventing this process by transcriptionally regulating anti-ferroptotic and antioxidant genes, which equilibrates iron/heme metabolism and reduces lipid peroxidation. Furthermore, we also observed an increased level of carbonylated proteins following 3 days of acute repeated stress. Previous studies demonstrated similar increases in hepatic protein oxidation following physiological stress employing acute exhaustive exercise [45,46]. In addition to protein carbonylation, we examined the effects of acute repeated stress on another form of post-translational protein modification caused by higher concentrations of nitrating species. The increase in 3-NT following 3 days of repeated stress represents a footprint of peroxynitrite formation and increased mitochondrial superoxide formation. Interestingly, Zhu and colleagues [47] demonstrated that Cu, Zn-SOD activity is crucial for the catalysis of hepatic protein nitration in murine models of metabolic stress. It is well established that hepatotoxic agents including lipopolysaccharide and acetaminophen induce excessive tyrosine-nitrated proteins accompanied by a marked up-regulation of inducible nitric oxide synthase expression [48,49,50,51]. In our stress model, we showed that there was also an up-regulation of inducible and endothelial NOS following a single episode of stress. Similar effects were observed in our previous studies in the central nervous system [52,53]. Zhang and colleagues [54] reported that the inducible NOS inhibitor, L-iminoethyl-lysine, reverses metabolic stress-induced GSH depletion, lipid peroxidation, and 4-HNE accumulation in the rat liver. Conversely, using a human NOS3 adenoviral vector, overexpression of endothelial NOS increased levels of nitrotyrosine, markers of liver injury, and apoptosis in mice following ischemia/reperfusion [55]. The authors also suggested that the observed liver injury was likely due to NO-induced inhibition of mitochondrial respiration in the endothelial NOS-overexpressing mice. Together, these studies suggest that both the inducible and endothelial form of NOS are capable of producing excessive NO to cause the observed peroxynitrite-mediated 3-NT and 4-HNE formation.

Despite the observed stress-induced oxidative and nitrosative stress in the liver, the expression of transcription factor Nrf2 was robustly down-regulated. Immunofluorescence analysis revealed that Nrf2 was shuttled into the nucleus following 1 day of stress exposure, whereas no nuclear Nrf2 signal was observed at day 3. Nuclear Nrf2 is likely redirected from a pool of existing Nrf2 protein and may be responsible for the observed transient up-regulation of antioxidant enzymes, including *Sod* isoforms and *Gpx4*. Numerous studies previously demonstrated that covalent modification of Nrf2 by phosphorylation/dephosphorylation and acetylation/deacetylation modulates Nrf2 nuclear translocation/export and degradation [56,57]. The compelling observation of Nrf2 down-regulation in hepatic tissues was also demonstrated in a previous in vitro study [27]. Using HEK-293 cells, Kratschmar and colleagues [27] demonstrated that activation of GR under physiological concentrations of glucocorticoids significantly suppressed Nrf2 transactivation capacity. Furthermore, Nrf2 activity was reduced following cortisol or cortisone incubation in H4IIE cells transiently transfected with rat 11β-Hsd1, with either GR antagonist or 11β-Hsd1 inhibitor reversing this glucocorticoid-dependent inhibition. It was suggested that recruitment of corepressors including silencing mediator for retinoid and thyroid hormone receptors (SMRTs) and nuclear receptor corepressor (N-CoRs) may be responsible for this GR-steroid complex-mediated Nrf2 repression [58]. Conversely, although expression of the related transcription factor, Nrf1, showed a modest up-regulation following acute stress, this minor change is unlikely to translate into a biologically relevant response. The observed down-regulation of Nrf2 does not seem to be associated with the Keap1 repressor at the transcriptional level. However, accumulating studies have now demonstrated alternative, Keap1-independent mechanisms of Nrf2 regulation, including phosphorylation of Nrf2 by protein kinases, interactions with other protein partners, and epigenetic factors [59,60,61]. The expression of *Gclc*, one of the primary Nrf2-controlled antioxidant enzymes, was down-regulated at both time-points examined. As a rate-limiting catalyst in the first step of GSH biosynthesis, the down-regulation of Gclc indicates that there is potentially no compensation for the loss in GSH observed in the present study. Previous work observed similar effects where transcripts encoding both subunits of glutamate-cysteine ligase were robustly reduced in Nrf2 knockout fibroblasts and liver tissues [62]. Furthermore, we observed that *Gpx4* was paradoxically increased following a single episode of restraint stress. It was well documented that Gpx4 is essential to reduce lipid peroxides and acts as a central endogenous suppressor of ferroptosis [63,64,65]. While the observed up-regulation in hepatic *Gpx4* expression may offer some cellular protection, the induction is transient, relatively low, and does not offset the loss of GSH or accumulation of 3-NT and 4-HNE.

The expression of two other ARE-regulated phase II detoxifying enzymes, *Hmox1* and *Nqo1*, was strongly up-regulated in the liver following 1 and 3 days of restraint stress. Although both enzymes were been shown to be regulated by Nrf2, several studies reported alternative regulation by transcription factor Foxo1 which specifically induces Hmox1 gene transcription by direct DNA binding, independent of Nrf2 [66,67,68]. Wang and colleagues [69] further demonstrated that protein expression of both Hmox1 and Nqo1 were significantly up-regulated in Foxo1-overexpressing murine cells and attenuated high glucose-induced oxidative stress. The observed up-regulation in *Foxo1* expression in the present study may be an important alternative factor driving antioxidant gene transcription during a state of GR-steroid complex-mediated Nrf2 repression. Furthermore, emerging studies on the epigenetic regulator Brd4 showed a modulatory role on the Nrf2-Keap1-ARE signaling pathway [70,71,72]. Although the minor change in *Brd4* suggests this is not biologically relevant to stress regulation in the liver, knockdown of Brd4 was previously shown to robustly down-regulate both Hmox1 gene and protein expression in HEK293T cells [70]. Together, these findings provide insights into the alternative transcriptional regulatory network of the cellular response to stress-induced hepatic oxidative stress.

Stress-induced oxidative stress appears to generate a tissue specific response that in the liver, is characterized by a robust and antithetical reduction in the Nrf2-Keap1-ARE signaling pathway. This results in substantial reductions in GSH/cysteine thiol-mediated cellular antioxidant capacity and increased oxidative damage to lipids and proteins. Moreover, despite high levels of circulating glucocorticoids, local hepatic tissue levels of corticosterone are likely potentiated further by a systematic increased in bioavailability mediated by reduced protein binding and increased reactivation. Our findings indicate that the liver displays an unusual physiological response to restraint stress that appears to exacerbate oxidative stress likely by impairing the canonical Nrf2-Keap1-ARE signaling pathway and the tissue’s antioxidant capacity.

## Figures and Tables

**Table 1:** List of Taqman^TM^ gene expression assays used for quantification of mRNA levels.

Gene Symbol	Gene Name	RefSeq Accession Number	Assay ID (in Applied Biosystems)
***Brd4***	Bromodomain-containing protein 4	NM_001100903.1	Rn01535560_m1
***Cat***	Catalase	NM_012520.2	Rn00560930_m1
***Foxo1***	Forkhead box O1	NM_001191846.2	Rn01494868_m1
***Gclc***	Glutamate-cysteine ligase, catalytic	NM_012815.2	Rn00689046_m1
***Gpx1***	Glutathione peroxidase 1	NM_030826.3	Rn00577994_g1
***Gpx4***	Glutathione peroxidase 4	NM_001039849.2	Rn00820818_g1
***Hmox1***	Heme oxygenase 1	NM_012580.2	Rn00561387_m1
***Hsd11b1***	Hydroxysteroid 11-beta dehydrogenase 1	NM_017080.2	Rn00567167_m1
***Hsd11b2***	Hydroxysteroid 11-beta dehydrogenase 2	NM_017081.2	Rn04341420_g1
***Keap1***	Kelch-like ECH-associated protein 1	NM_057152.2	Rn01448220_m1
***Nfe2l1*** *(**Nrf1**)*	Nuclear factor, erythroid 2-like 1	XM_006247236.2	Rn01452824_m1
***Nfe2l2*** *(**Nrf2**)*	Nuclear factor, erythroid 2-like 2	NM_031789.2	Rn00582415_m1
***Nqo1***	NAD(P)H dehydrogenase, quinone 1	NM_017000.3	Rn00566528_m1
***Nos2***	Nitric oxide synthase 2, inducible	NM_012611.3	Rn00561646_m1
***Nos3***	Nitric oxide synthase 3, endothelial	NM_021838.2	Rn02132634_s1
***Nr3c1***	Nuclear receptor subfamily 3, group C, member 1	NM_012576.2	Rn00561369_m1
***Serpina6*** *(**Cbg**)*	Serpin peptidase inhibitor, clade A (alpha-1 antiproteinase, antitrypsin), member 6	NM_001009663.1	Rn01517119_m1
***Sod1***	Superoxide dismutase 1, soluble	NM_017050.1	Rn00566938_m1
***Sod2***	Superoxide dismutase 2, mitochondrial	NM_017051.2	Rn00690588_g1
